# Persistent primitive hypoglossal artery associated with Chiari II malformation: Diagnosis and clinical implications

**DOI:** 10.4103/0971-3026.73534

**Published:** 2010-11

**Authors:** Mudit Gupta, Rashmi Gupta, Ashu Seith

**Affiliations:** Department of Radiodiagnosis, All India Institute of Medical Sciences, Ansari Nagar, New Delhi - 110 029, India

**Keywords:** Chiari malformation, hypoglossal, meningocele

## Abstract

We present a case of persistent primitive hypoglossal artery (PPHA) associated with Chiari II malformation and discuss the clinical implications. There has been one reported case of PPHA associated with Chiari 1 malformation, but none in association with Chiari II. Our patient also had a widened hypoglossal canal, with cerebrospinal fluid (CSF) sac herniation through it.

## Introduction

A case of PPHA with coexistant Chiari II and a meningocele passing through the hypoglossal canal is presented. This combination is very unusual. Imaging features with clinical and management implications of this entity are described.

## Case Report

A 14-day-old male baby presented to the Pediatric Outpatient Department with a low-back mass since birth. A clinical diagnosis of meningocele / meningomyelocele was made. The patient was referred for MRI, to see the contents of the sac and for imaging of the brain.

**Figure 1 (A, B):**
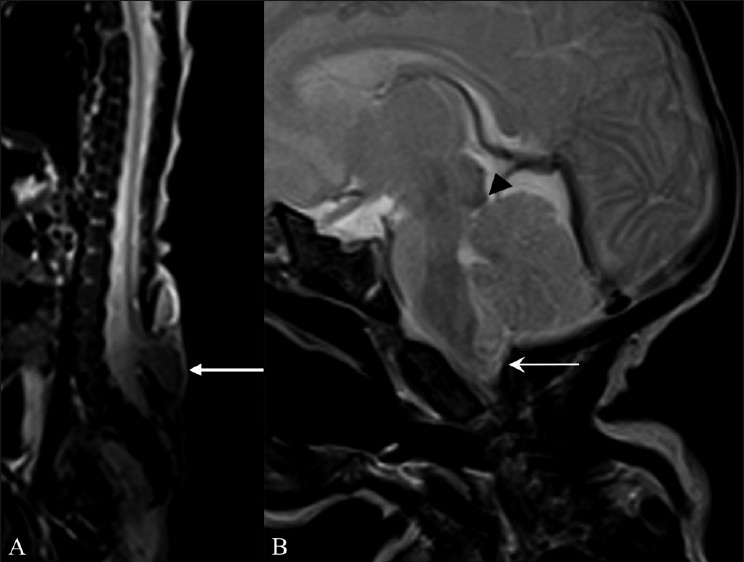
Sagittal T2W whole spine MRI (A) shows a meningomyelocele (arrow) opposite the L5 and S1 vertebrae. Sagittal T2W MRI of the brain (B) shows a small posterior cranial fossa, with herniation of the cerebellar vermis and tonsils (arrow) through the foramen magnum with tectal beaking (arrowhead). These findings are suggestive of an Arnold–Chiari II malformation

**Figure 2 (A, B):**
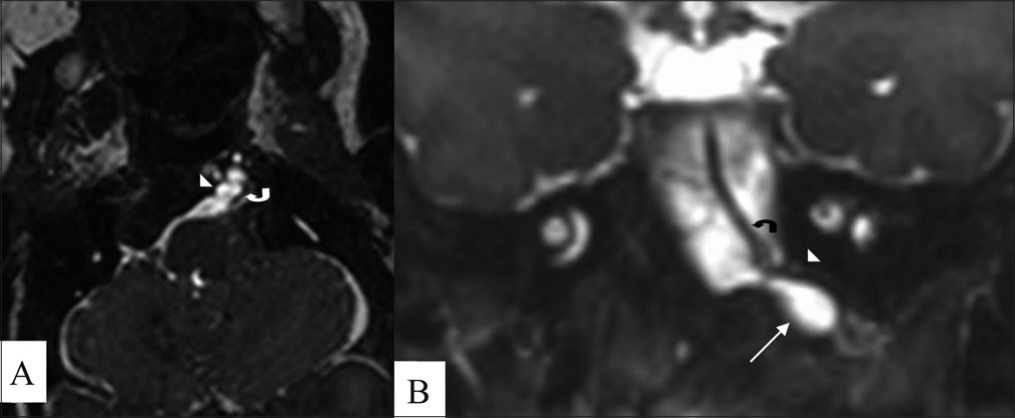
Axial (A) and coronal reformatted (B) constructive interference steady state (CISS) MRI images show herniation of a CSF sac (arrow) through a deficient left hypoglossal canal (arrowhead); there is also a vascular channel (curved arrow) passing through the canal

**Figure 3:**
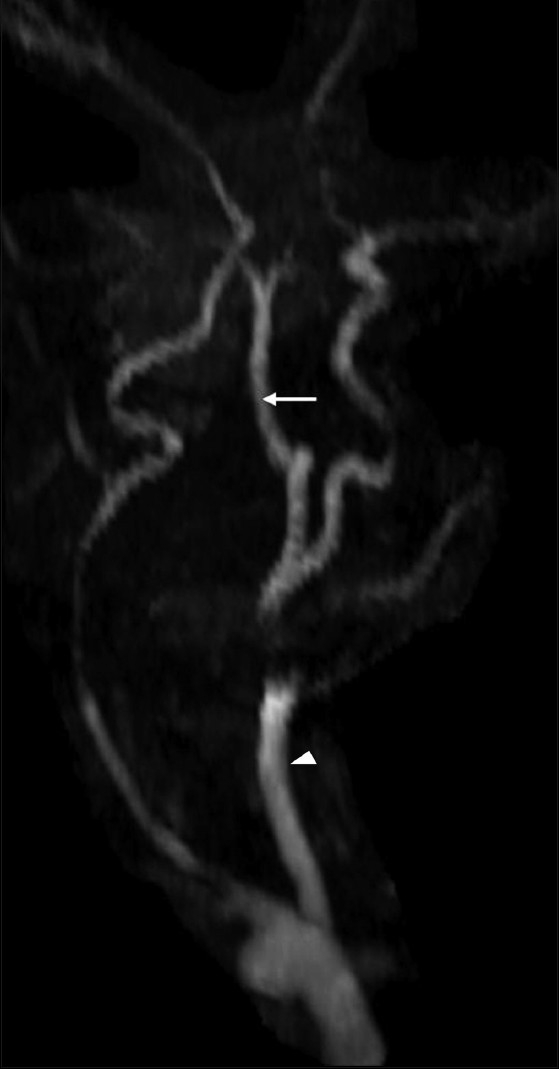
Time of flight MRI angiography shows absence of both vertebral arteries with a persistent primitive hypoglossal artery (white arrow) arising from the left internal carotid artery (arrowhead)

On MRI, a meningomyelocele was seen in the back opposite the L5 and S1 vertebrae [Figure 1A]. Imaging of the brain revealed an Arnold–Chiari malformation, type II [Figure 1B]. In addition, there was herniation of the CSF space of the premedullary cistern through a deficiency of the hypoglossal canal anteriorly, into the retropharyngeal space. There were no neural elements seen within. This lesion was seen displacing the internal carotid artery (ICA) anterolaterally. Another tubular vascular structure was seen coursing along with the CSF space [Figures 2A, B]. A time-of-flight (TOF) MRI angiography (MRA) and contrast-enhanced MRA were performed with maximum-intensity projections (MIP). An artery was seen arising from the left *internal carotid artery* (ICA) and passing through the left hypoglossal canal and the premedullary cistern space, to join the basilar artery; this was suggestive of PPHA. The vertebral arteries were not visualized bilaterally [Figure 3].

Hence, a final diagnosis of PPHA with Arnold–Chiari II malformation with CSF herniation through the hypoglossal canal was made. Closure and repair was done for the lumbar meningomyelocele. The CSF sac herniation through the hypoglossal canal was not repaired due to the high surgical risk associated with the presence of a PPHA.

## Discussion

**Figure 4:**
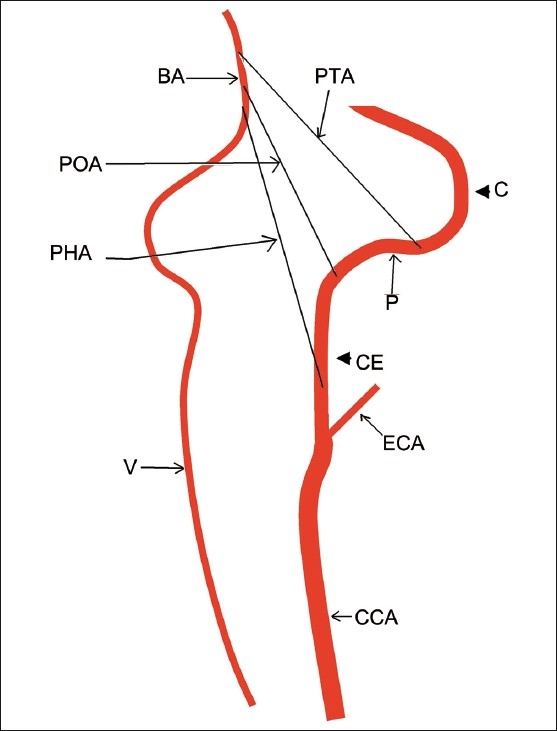
Line diagram illustrates various persistent carotid–basilar anastomoses. PTA = persistent trigeminal artery, PHA = persistent hypoglossal artery, POA = persistent otic artery, BA = basilar artery, V = vertebral artery, CCA = common carotid artery, ECA = external carotid artery, CE = cervical course of internal carotid artery (ICA), P = petrous course of ICA, C = cavernous course of ICA. The PTA joins the ICA just below the cavernous part, while the POA joins it just below the petrous part, whereas, the PHA joins the cervical part of the ICA

During the early part of embryogenesis, various anastomoses exist between the primitive ICA and the vertebrobasilar system. These anastomotic arteries include primitive trigeminal, primitive hypoglossal, and primitive otic arteries [Figure 4]. These vessels disappear with the development of the vertebral arteries. Failure of involution results in the persistence of these fetal arteries, often accompanied by improper development of the vertebral arteries. These persistent fetal arteries maintain the posterior circulation. Most common among these are a persistent trigeminal artery, with a reported incidence of 0.1 – 0.2%, followed by a persistent hypoglossal artery, with a reported incidence of 0.027 – 0.26%.[1]

The persistent primitive hypoglossal artery is a type of persistent carotid-basilar anastomosis.[2] This anomalous vessel courses through the hypoglossal canal and parallels a part of the course of the hypoglossal nerve, to join the cervical ICA along with the basilar artery.[3] It is known to be associated with intracranial aneurysms,[4] arteriovenous malformations, and stroke. There has been only one reported case of its association with Chiari I,[5] but an association with Chiari II has never been reported.

Coexistence of PPHA with Chiari II will further worsen the prognosis for this patient. Inappropriate development of the vertebral arteries in these patients means that the PPHA represents the only vascular channel supplying posterior circulation.[6] Any insult to this artery would be detrimental to posterior circulation. Persistence of these arteries is commonly diagnosed during percutaneous angiographic procedures. Diagnosis of PPHA by noninvasive modalities such as CT angiography or MRA would reduce the chances of iatrogenic injury.

The CSF sac herniation through the hypoglossal canal probably represents an unusual type of meningocele. In literature there have been isolated reports of many rare types of meningoceles, such as those protruding through the sphenoid bone as well as transclival or transethmoidal meningoceles.[7,8] However, after an extensive literature search we found no report of a meningocele passing through the hypoglossal canal.

Cranial meningoceles are commonly associated with complications such as intracranial infection and epilepsy, and hence, surgical management is required. Coexistence of a primitive hypoglossal artery increases the chances of surgical complications and danger to the posterior circulation.

A combination of PPHA, Chiari II malformation, and a meningocele passing through the hypoglossal canal, to the best of our knowledge, has never been reported in literature. This combination has significant prognostic and surgical implications.
